# *NEDD9*, an independent good prognostic factor in intermediate-risk acute myeloid leukemia patients

**DOI:** 10.18632/oncotarget.18537

**Published:** 2017-06-16

**Authors:** Victor Pallarès, Montserrat Hoyos, M. Carmen Chillón, Eva Barragán, M. Isabel Prieto Conde, Marta Llop, María Virtudes Céspedes, Josep F. Nomdedeu, Salut Brunet, Miguel Ángel Sanz, Marcos González-Díaz, Jorge Sierra, Isolda Casanova, Ramon Mangues

**Affiliations:** ^1^ Biomedical Research Institute Sant Pau (IIB Sant Pau), Hospital de la Santa Creu i Sant Pau, Barcelona, Spain; ^2^ Department of Hematology, Hospital de la Santa Creu i Sant Pau, Barcelona, Spain; ^3^ Department of Hematology, University Hospital of Salamanca and Center of Investigation in Cancer-IBMCC, Instituto de Investigación Biomédica de Salamanca (IBSAL), Salamanca, Spain; ^4^ Clinical Laboratory and Department of Hematology, University Hospital La Fe, Valencia, Spain; ^5^ CIBER en Bioingeniería, Biomateriales y Nanomedicina (CIBER-BBN), Barcelona, Spain; ^6^ Josep Carreras Leukemia Research Institute, Barcelona, Spain; ^7^ Universitat Autònoma de Barcelona, Barcelona, Spain; ^8^ CIBER in Oncology (CIBER-ONC), Madrid, Spain

**Keywords:** acute myeloid leukemia, *nedd9*, bcar1, prognostic factor, intermediate-risk

## Abstract

Intermediate-risk acute myeloid leukemia (IR-AML) is the largest subgroup of AML patients and is highly heterogeneous. Whereas adverse and favourable risk patients have well-established treatment protocols, IR-AML patients have not. It is, therefore, crucial to find novel factors that stratify this subgroup to implement risk-adapted strategies. The CAS (Crk-associated substrate) adaptor protein family regulates cell proliferation, survival, migration and adhesion. Despite its association with metastatic dissemination and prognosis of different solid tumors, the role of these proteins in hematological malignancies has been scarcely evaluated. Nevertheless, previous work has established an important role for the CAS family members NEDD9 or BCAR1 in the migratory and dissemination capacities of myeloid cells. On this basis, we hypothesized that NEDD9 or BCAR1 expression levels could associate with survival in IR-AML patients and become new prognostic markers. To that purpose, we assessed *BCAR1* and *NEDD9* gene expression in a cohort of 73 adult AML patients validating the results in an independent cohort (*n* = 206). We have identified *NEDD9*, but not *BCAR1*, as a new a marker for longer overall and disease-free survival, and for lower cumulative incidence of relapse. In summary, *NEDD9* gene expression is an independent prognostic factor for favourable prognosis in IR-AML patients.

## INTRODUCTION

Acute myeloid leukemia (AML) is a heterogeneous group of diseases characterized by the infiltration of bone marrow, blood, and other tissues by proliferative, clonal and abnormally differentiated immature cells of the hematopoietic system [1–3].

According to cytogenetic and molecular features, AML patients are classified into favorable, intermediate, or adverse risk groups and their therapy after remission are risk-adapted [2]. Although many favorable and adverse group patients are well characterized by specific chromosomal alterations, the intermediate-risk cytogenetics group (IR-AML) is heterogeneous and includes patients with a widely diverse outcome. In contrast to established treatment protocols for patients in the favorable and adverse risk groups, the clinical decision guideline for the IR-AML group remains unclear [4]. This uncertainty regarding therapy impairs survival of many IR-AML patients. Therefore, there is a need to find new prognostic factors to stratify this group of patients allowing clinicians to choose better treatments and improve their outcome. For that reason, several genetic alterations have been evaluated over the last years in an attempt to improve patient stratification of the IR-AML group. At present, only the molecular markers NPM1, CEBPA and FLT3 have been incorporated into clinical practice [2, 5, 6]. For instance, a combination of mutated NPM1 and non-mutated FLT3/Internal tandem duplication (FLT3/ITD) associates with favorable outcome in IR-AML patients [7–10]. Similarly, biallelic CEBPA mutations predict a relatively favorable outcome in patients without FLT3/ITD [11]. Recently, mutations of RUNX1, ASXL1 and TP53 have been associated with adverse outcome and are also included in the standard diagnosis [4]. Additional molecular factors, such as IDH1, IDH2, TET2, DNMT3A, MLL-PTD or NRAS, have demonstrated capacity to stratify a subset of IR-AML patients, allocating to them into the good or poor prognosis groups [12, 13]; nevertheless, they have not been introduced in clinical practice yet; therefore the need still remains to identify new prognostic indicators with higher stratification capacity.

The Crk-Associated Substrate (CAS) is a family of focal adhesion related proteins that play crucial roles in the regulation of cell growth, adhesion, migration and apoptosis [14]. They are involved in signal transduction downstream of integrin engagement and receptor tyrosine kinases (RTKs), which signal through the FAK, SRC and CAS family proteins. In this context, upregulation of scaffolding proteins such as NEDD9 or BCAR1, and also of the FAK and SRC protein kinases, has shown pro-metastatic capacity in different solid tumors and associates with poor outcome [14, 15]. The role of focal adhesion proteins in hematologic malignancies has been less studied. In this regard, FAK and SRC are prognostic factors that associate with poor outcome in AML [16, 17]. In the present study, we investigated whether *NEDD9* and *BCAR1* could have prognostic significance and correlate with survival in IR-AML patients. We have found, in two independent patient cohorts, the expression of *NEDD9*, but not *BCAR1*, to be an independent prognostic factor in IR-AML patients, that associates with better outcome.

## RESULTS

### Patient characteristics

The main characteristics of the patients from the two cohorts are shown in Table 1. In summary, the Cohort 1 had 73 patients, while the Cohort 2 had 206 patients. The median ages were 52 and 53 years with a 47% and a 43% of patients younger than 50 years, respectively. Most of the characteristics, including age, gender, NPM1 and FLT3/NPM1 combined mutations did not show significant differences between the two cohorts. However, in Cohort 1, the median of white blood cells (WBC) count, the percentage of patients with the WBC count over 20 × 10^9^/L, FLT3/ITD^+^ and CEBPA^-^ were significantly higher than in Cohort 2. The percentage of patients in some subgroups of the French-American-British (FAB) classification was also significantly different between cohorts.

**Table 1:** Patient's characteristics and comparison between the cohorts of our study

Parameter	Cohort 1	Cohort 2	*P*
	(*n* = 73)	(*n* = 206)	
Age, median (range)	52 (17–65)	53 (17–65)	0.187^†^
< 50 years (%)	34 (47)	88 (43)	0.585
> 50 years (%)	39 (53)	118 (57)	
Sex,			
Male (%)	39 (53)	109 (53)	1.000
Female (%)	34 (47)	97 (47)	
WBC, median (range)	60 (2–325)	16 (0–324)	**< 0.001**^‡^
< 20 × 10^9^/L (%)	25 (34)	107 (53)	**0.009**
> 20 × 10^9^/L (%)	48 (66)	97(47)	
FAB Classification (%)			0.045*
M0	8 (11)	16 (8)	
M1	23 (32)	47 (23)	
M2	6 (8)	41 (20)	
M4	12 (16)	37 (18)	
M4E	1 (1)	1 (1)	
M5	21 (29)	46 (22)	
M6	1 (1)	6 (3)	
M7	1 (1)	0 (0)	
Unknown	0 (0)	12 (6)	
Molecular alterations (%)			
FLT3/ITD^−^	39 (53)	149 (72)	**0.023**
FLT3/ITD^+^	29 (40)	56 (27)	
Unknown	5 (7)	1(1)	
NPM1^−^	31 (43)	108 (52)	0.570
NPM1^+^	33 (45)	97 (47)	
Unknown	9 (12)	1 (1)	
FLT3^−^ + NPM1^+^(Favorable)	16(22)	60(29)	0.633
Others (Unfavorable)	47(64)	145(70)	
Unknown	10(14)	1(1)	
CEBPA^−^	57(78)	78(38)	< 0.001*
CEBPA^+^	1(1)	11(5)	
Unknown	15(21)	117(57)	

Results are presented as the number of patients for each characteristic. The percentage of the patients is indicated in brackets for each condition. For categorical variables, the Fischer exact test or χ^2^ test (*) were used to analyze the statistical significance; while for continuous variables showing normal or abnormal distribution, the Student's *t*-test (†) or the Mann-Whitney U (‡) test were used, respectively. *P* < 0.05 indicates statistical significance (Bold values). WBC; White blood cells. FAB; French-American-British.

Clinical outcomes such as overall survival (OS) and disease-free survival (DFS) between the two cohorts did not present statistically significant differences (OS, *p* = 0.610; DFS, *p* = 0.904) (Data not shown).

The alive patients had a median follow-up of 56 and 58 months in the Cohort 1 and 2, respectively, and the OS at 5 years for the patients of the two groups were 47.0 ± 6.1 % and 48.8 ± 3.7 %, respectively (Supplementary Table 1).

After a median follow-up for patients alive or in complete remission (CR) of 54 months in Cohort 1 and 46 months in Cohort 2, 29% (17/59) and 35% (64/185) of patients in Cohorts 1 and 2 respectively, relapsed (Supplementary Table 1). At 5 years, DFS for patients of two groups were 51.4 ± 6.6 and 44.8 ± 4.1 % and cumulative incidence of relapse (CIR) were 29.6 ± 6.1 and 37.8 ± 3.9 %, respectively. None of the variables analyzed (age, FLT3/ITD duplication and FLT3/NPM1 combined mutations) in Cohort 1 had an impact on DFS or CIR. In contrast, in Cohort 2, patients older than 50 years, with FLT3/ITD mutation or with the unfavorable FLT3/NPM1 combination (FLT3^+^/NPM1^−^, FLT3^−^/NPM1^−^ and FLT3^+^/NPM1^+^) showed the worst survival after a CR and a higher incidence of relapse than younger patients, patients without FLT3/ITD or with a favorable FTL3/NPM1 combination (FLT3^−^/NPM1^+^) (Supplementary Table 1).

Furthermore, 52% (38/73) and 49% (101/206) of patients in Cohort 1 and 2, respectively, died (Supplementary Table 1).

### *NEDD9* is an independent prognostic factor for OS and DFS in IR-AML patients

Clinical variables as age, sex, WBC, FLT3/ITD duplication, NPM1 mutation and FLT3/NPM1 combined mutations, as well as *BCAR1* and *NEDD9* expression were assessed in the univariate analysis. The variables with a *p-*value higher than 0.25 were not included in the multivariate analysis.

In order to establish the cutoff for *BCAR1* and *NEDD9* expression, we performed ROC (Receiver Operating Characteristic) curves. However, we could not find any point with enough specificity and sensitivity. Thus, we decided to perform exploratory univariate analyses using the mean, the median or the quartiles as thresholds. In *BCAR1* analyses, no statistically significant difference was found with any cutoff. On the contrary, when we used the mean as threshold we found *NEDD9* as a good prognostic factor of OS in Cohort 1 (*p* = 0.003) and DFS in the two cohorts (Cohort 1 *p* = 0.019 and Cohort 2 *p* = 0.046). Using the median as threshold, *NEDD9* expression was significant in two clinical outcome endpoints (OS *p* = 0.009 and DFS *p* = 0.003) in the Cohort 1, in contrast, in the Cohort 2 it was not in any of them. Finally, when we established the third quartile as the cutoff we found the best results in both cohorts, so we decided to perform all the analysis using the third quartile to define overexpression of *NEDD9*.

In the univariate analysis of Cohort 1 (Tables 2 and 3), Cox regression and Fine and Gray's tests showed that age over 50 years and *NEDD9* overexpression (over the third quartile) were associated with lower and higher OS, respectively (*p* = 0.031, Hazard Ratio (HR) = 2.071; *p* = 0.026, HR = 0.343, resp.). Regarding DFS, only *NEDD9* expression showed a trend towards significance (*p* = 0.067) while any variable was significant in the CIR univariate analyses. In Cohort 2 analyses, *NEDD9* expression (overexpression), and FLT3/NPM1 combination (unfavorable combinations: FLT3^+^/NPM1^−^, FLT3^−^/NPM1^−^ and FLT3^+^/NPM1^+^) had significant differences in OS (*p* = 0.029, HR = 0.566; *p* = 0.002, HR = 2.243, resp.), DFS (*p* = 0.006, HR=0.468; *p* = 0.002, HR = 2.229, resp.) and CIR (*p* = 0.040, HR=0.519; *p* = 0.016, HR = 2.030, resp.) studies. Moreover, age (> 50 years) in OS and DFS (*p* = 0.008, HR = 1.761; *p* = 0.038, HR = 1.555, resp.), NPM1 (NPM1^+^) in OS (*p* = 0.045, HR = 0.658) and FLT3/ITD duplication (FLT3/ITD^+^) in DFS and CIR (*p* = 0.025, HR = 1.636; *p* = 0.016, HR = 1.720, resp.) were also statistically significant. *BCAR1* expression had no differences in any univariate analyses of any cohort.

Table 2Univariate and multivariate analyses of *NEDD9*, *BCAR1* and clinical variables in OS and DFS

**Table:** 

		Cohort 1 (*n* = 73)						Cohort 2 (*n* = 204)					
OS		Univariate			Multivariate			Univariate			Multivariate		
Variable	Item	HR	95% CI	*P* value	HR	95% CI	*P* value	HR	95% CI	*P* value	HR	95% CI	*P* value
Age	< 50 years	1						1					
	> 50 years	2.071	(1.067–4.018)	**0.031**	2.542	(1.290–5.011)	0.007	1.761	(1.159–2.676)	**0.008**	2.011	(1.311–3.085)	**0.001**
Sex	Male	1						1					
	Female	1.701	(0.898–3.221)	0.103	1.636	(0.859–3.116)	0.134	0.808	(0.543–1.202)	0.292	–	–	–
WBC	< 20 × 10^9^/L	1						1					
	> 20 × 10^9^/L	0.709	(0.370–1.361)	0.301	–	–	–	1.019	(0.685–1.515)	0.926	–	–	–
FLT3	FLT3/ITD^–^	1						1					
	FLT3/ITD^+^	1.071	(0.555–2.067)	0.839	–	–	–	1.315	(0.851–2.031)	0.217	1.080	(0.541–2.153)	0.828
NPM1	NPM1^–^	1						1					
	NPM1^+^	1.252	(0.628–2.495)	0.523	–	–	–	0.658	(0.438–0.991)	**0.045**	1.112	(0.513–2.408)	**0.788**
FLT3/NPM1	Favorable	1						1					
	Unfavorable	1.222	(0.527–2.832)	0.640	–	–	–	2.243	(1.344–3.745)	**0.002**	2.686	(1.047–6.889)	**0.040**
NEDD9	Underexpression	1						1					
	Overexpression	0.343	(0.134–0.883)	**0.026**	0.243	(0.085–0.698)	**0.009**	0.566	(0.339–0.944)	**0.029**	0.582	(0.341–0.995)	**0.048**
BCAR1	Underexpression	1						1					
	Overexpression	0.765	(0.330–1.773)	0.532	–	–	–	0.925	(0.569–1.506)	0.755	–	–	–

**Table:** 

		Cohort 1 (*n* = 59)						Cohort 2 (*n* = 185)					
DFS		Univariate			Multivariate			Univariate			Multivariate		
Variable	Item	HR	95% CI	*P* value	HR	95% CI	*P* value	HR	95% CI	*P* value	HR	95% CI	*P* value
Age	< 50 years	1						1					
	> 50 years	1.642	(0.782–3.448)	0.191	2.053	(0.962–4.381)	0.063	1.555	(1.024–2.360)	**0.038**	1.845	(1.203–2.829)	**0.005**
Sex	Male	1						1					
	Female	1.276	(0.615–2.646)	0.513	–	–	–	0.853	(0.570–1.279)	0.442	–	–	–
WBC	< 20 × 10^9^/L	1						1					
	< 20 × 10^9^/L	0.695	(0.332–1.455)	0.335	–	–	–	1.041	(0.694–1.561)	0.846	–	–	–
FLT3	FLT3/ITD^–^	1						1					
	FLT3/ITD^+^	0.914	(0.424–1.971)	0.818	–	–	–	1.636	(1.063–2.519)	**0.025**	1.606	(0.998–2.584)	0.051
NPM1	NPM1^–^	1						1					
	NPM1^+^	1.369	(0.596–3.143)	0.459	–	–	–	0.793	(0.528–1.193)	0.266	–	–	–
FLT3/NPM1	Favorable	1						1					
	Unfavorable	1.247	(0.490–3.176)	0.643	–	–	–	2.229	(1.345–3.693)	**0.002**	2.079	(1.203–3.595)	**0.009**
*NEDD9*	Underexpression	1						1					
	Overexpression	0.372	(0.129–1.072)	0.067	0.304	(0.103–0.893)	**0.030**	0.468	(0.273–0.803)	**0.006**	0.426	(0.241–0.753)	**0.003**
*BCAR1*	Underexpression	1						1					
	Overexpression	0.782	(0.290–2.111)	0.628	–	–	–	0.947	(0.573–1.562)	0.830	–	–	–

COX test was used to analyze the statistical significance in OS and DFS. *P* value < 0.05 was considered statistically significant (Bold values). “-“ indicates that variables were not included in the multivariate analyses (*P* value > 0.250 in the univariate analysis). HR; Hazard ratio. CI; Confidence interval. OS; Overall survival. DFS, Disease-free survival. WBC; White blood cells.

**Table 3:** Univariate and multivariate analyses of *NEDD9*, *BCAR1* and clinical variables in CIR

		Cohort 1 (*n* = 59)						Cohort 2 (*n* = 185)					
CIR		Univariate			Multivariate			Univariate			Multivariate		
Variable	Item	HR	95% CI	*P* value	HR	95% CI	*P* value	HR	95% CI	*P* value	HR	95% CI	*P* value
Age	< 50 years	1						1					
	> 50 years	1.160	(0.453–2.970)	0.760	–	–	–	1.080	(0.656–1.770)	0.770	–	–	–
Sex	Male	1						1					
	Female	0.842	(0.326–2.180)	0.720	–	–	–	1.030	(0.634–1.680)	0.900	–	–	–
WBC	< 20 × 10^9^/l	1						1					
	>20 × 10^9^/l	2.110	(0.710–6.250)	0.180	2.080	(0.623–6.940)	0.230	1.090	(0.672–1.780)	0.720	–	–	–
FLT3	FLT3/ITD^−^	1						1					
	FLT3/ITD^+^	1.300	(0.714–2.370)	0.390	–	–	–	1.720	(1.100–2.680)	0.016	1.660	(0.911–3.024)	0.098
NPM1	NPM1^−^	1						1					
	NPM1^+^	0.875	(0.456–1.680)	0.690	–	–	–	0.814	(0.501–1.320)	0.410	–	–	–
FLT3/NPM1	Favorable	1						1					
	Unfavorable	6.360	(0.903–44.700)	0.063	6.050	(0.870–42.050)	0.069	2.030	(1.140–3.620)	0.016	1.615	(0.855–3.052)	0.140
*NEDD9*	Underexpression	1						1					
	Overexpression	0.606	(0.185–1.990)	0.410	–	–	–	0.519	(0.278–0.969)	0.040	0.441	(0.220–0.884)	0.021
*BCAR1*	Underexpression	1						1					
	Overexpression	0.578	(0.134–2.490)	0.460	–	–	–	0.767	(0.409–1.440)	0.410	–	–	–

Gray and Fine and Gray tests for CIR were used to analyze the statistical significance. *P* value < 0.05 was considered statistically significant (Bold values). “–“ indicates that variables were not included in the multivariate analyses (*P* value > 0.250 in the univariate analysis). HR; Hazard ratio. CI; Confidence interval. CIR; Cumulative incidence of relapse. WBC; White blood cells.

In multivariate analyses for *NEDD9* (Tables 2 and 3), all of these variables kept their statistical significance except for NPM1 mutation in OS, FLT3/ITD duplication in DFS and CIR, and FLT3/NPM1 combined mutations in CIR of Cohort 2. Moreover, in Cohort 1 study, although the *NEDD9* expression had a *p-*value of 0.067 in the univariate analysis in DFS (HR = 0.372), it showed significant differences in the multivariate analysis (*p* = 0.030, HR = 0.304).

In all cases with statistical significance, age over 50 years, FLT3/NPM1 unfavorable combinations and FLT3/ITD duplication had a HR above 1, whereas the HR of *NEDD9* overexpression and NPM1 mutation was lower than 1.

### Survival curves

**Figure 1:**
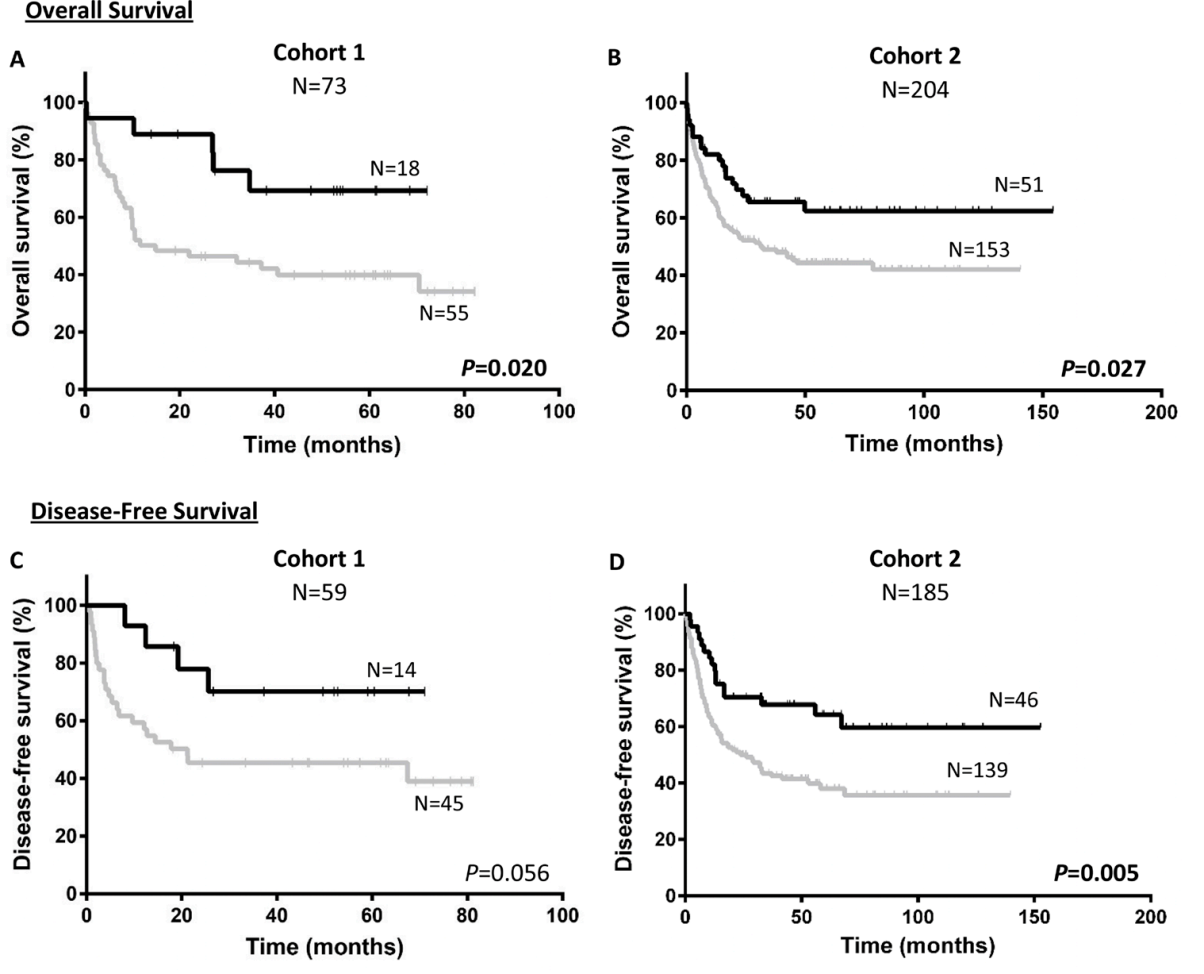
Survival according to *NEDD9* expression in cohorts Kaplan–Meier curves represent OS of cohort 1 (**A**) and cohort 2 (**B**), and DFS of cohort 1 (**C**) and cohort 2 (**D**) depending on the *NEDD9* expression. Black line and gray line indicate *NEDD9* overexpressed and underexpressed, respectively. Log-rank test was used to analyze the statistical significance. *P* < 0.05 was considered statistically significant (Bold values). OS; Overall survival. DFS; Disease-free survival.

**Figure 2:**
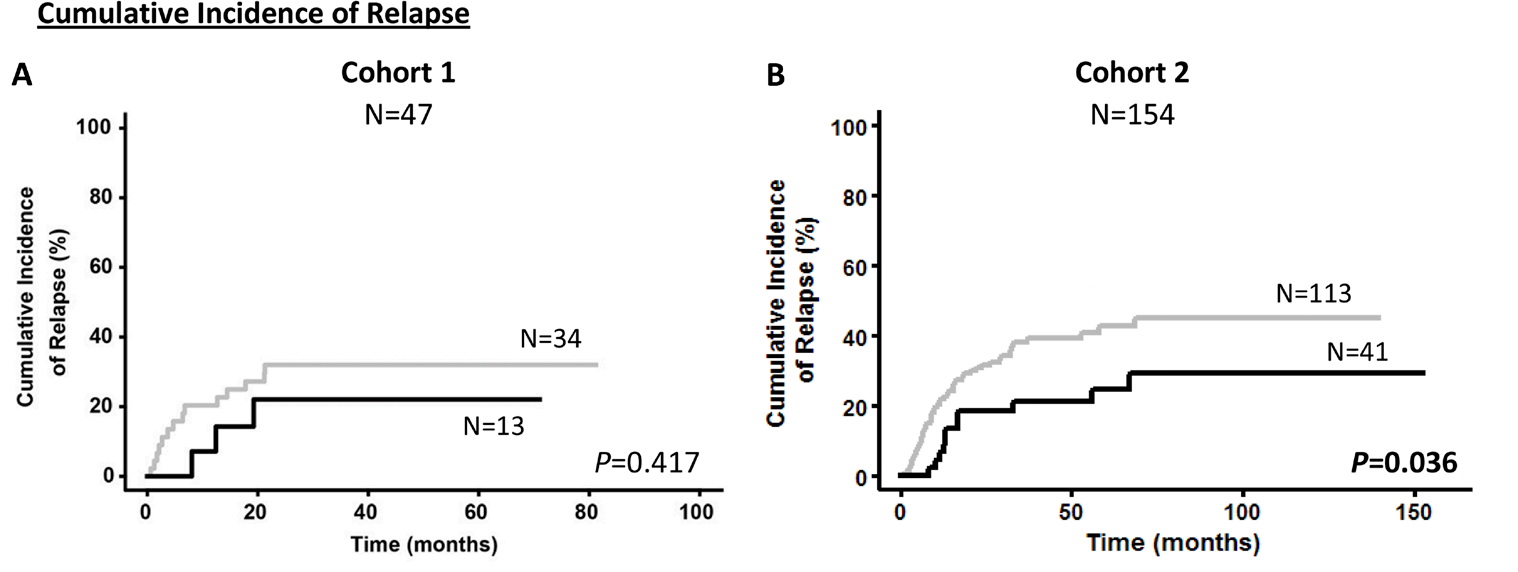
Cumulative incidence of relapse according to *NEDD9* expression in cohorts Kaplan–Meier curves represent CIR of cohort 1 (**A**) and cohort 2 (**B**) depending on the *NEDD9* expression. Black line and gray line indicate *NEDD9* overexpressed and underexpressed, respectively. Gray test for CIR was used to analyze the statistical significance. *P* < 0.05 was considered statistically significant (Bold values). CIR; Cumulative incidence of relapse.

Kaplan–Meier analysis showed that patients with overexpression of *NEDD9* (over the third quartile) had better prognosis than patients with lower expression of *NEDD9* (Figures 1 and 2). Significantly, the Cohort 1 patients with overexpression of *NEDD9* had an increase in OS (69.3 ± 11.6 vs 34.2 ± 7.9%, *p* = 0.020) (Figure 1), which was validated by the Cohort 2 analysis (62.4 ± 7.1 vs 42.1 ± 4.6%, *p* = 0.027). In addition, in the Cohort 1 patients with *NEDD9* overexpression showed a clear trend towards significance in predicting better DFS (70.1 ± 12.6 vs 39.0 ± 8.8%, *p* = 0.056) (Figure 1). Indeed, this difference reached significance in the Cohort 2 due to its higher number of patients (59.6 ± 8.3 vs 35.6 ± 5.0%, *p* = 0.005). In a similar way, although the differences between patients that overexpressed and underexpressed *NEDD9* in the Cohort 1 were not statistically significant in CIR analyses (22.1 ± 11.8 vs 32.0 ± 7.2%, *p* = 0.417), the same study in Cohort 2 presented significant differences (CIR: 29.2 ± 8.0 vs 45.0 ± 5.2%, *p* = 0.036) (Figure 2). No differences were found in the Kaplan–Meier analyses of *BCAR1* expression (Supplementary Figure 1).

### Association of *NEDD9* expression with clinical and molecular characteristics of AML patients

*NEDD9* association with several variables such as age, sex, WBC, FAB classification, FLT3/ITD duplication, NPM1 mutation, FLT3/NPM1 combined mutations, disease status (relapsed or no relapsed) and patient status at the end of follow up (alive or death) was evaluated in each cohort. Most of the variables analyzed had no association with the *NEDD9* expression (Table 4). Only FAB classification and the patient status at the end of the study (alive or death) showed significant association with *NEDD9* expression.

**Table 4:** Association between *NEDD9* expression and clinical data in the cohorts of our study

Parameter	Cohort 1	*NEDD9*			Cohort 2	*NEDD9*		
	Total (*n* = 73)	Under-expressed	Over-expressed	*p*-value	Total (*n* = 204)	Under-expressed	Over-expressed	*p*-value
Age,	73	55	18	0.726†	204	153	51	0.405†
< 50 years (%)	34 (47)	27	7	0.588	87 (43)	63	24	0.514
> 50 years (%)	39 (53)	28	11		117 (57)	90	27	
Sex,								
Male (%)	39 (53)	29	10	1.000	108 (53)	87	21	0.074
Female (%)	34 (47)	26	8		96 (47)	66	30	
WBC,	73	55	18	0.774‡	202	151	51	0.818‡
<20 × 10^9^/L (%)	25 (34)	18	7	0.776	107 (53)	82	25	0.521
>20 × 10^9^/L (%)	48 (66)	37	11		95 (47)	69	26	
FAB Classification, (%)				0.026*				0.029*
M0	8 (11)	7	1		16 (8)	14	2	
M1	23 (32)	21	2		46 (22)	39	7	
M2	6 (8)	6	0		41 (20)	33	8	
M4	12 (16)	8	4		36 (18)	28	8	
M4E	1 (1)	1	0		1 (1)	1	0	
M5	21 (29)	10	11		46 (22)	27	19	
M6	1 (1)	1	0		6 (3)	5	1	
M7	1 (1)	1	0		0 (0)	0	0	
Protein mutations, (%)								
FLT3/ITD^−^	39 (53)	30	9	1.000	149 (73)	116	33	0.198
FLT3/ITD^+^	29 (40)	22	7		54 (27)	37	17	
NPM1^−^	31 (43)	22	9	0.779	108 (53)	85	23	0.257
NPM1^+^	33 (45)	25	8		95 (47)	68	27	
FLT3^−^ + NPM1^+^ (Fav.)	16 (22)	12	4	1.000	60 (30)	43	17	0.476
Others (Unfav.)	47 (64)	35	12		143 (70)	110	33	
Alive (%)	35 (48)	22	13	**0.028**	105 (51)	72	33	**0.035**
Death (%)	38 (52)	33	5		99 (49)	81	18	
No relapse (%)	42 (71)	31	11	0.737	129 (67)	93	36	0.152
Relapse (%)	17 (29)	14	3		63 (33)	52	11	

Results are presented as the number of patients for each characteristic. The percentage of the patients is indicated in brackets for each condition. For categorical variables, the Fischer exact test or χ^2^ test (*) were used to analyze the statistical significance; while for continuous variables showing normal or abnormal distribution, the Student's *t*-test (†) or the Mann-Whitney *U* (‡) test were used, respectively. *P* < 0.05 indicates statistical significance (Bold values). WBC; White blood cells. FAB; French-American-British.

## DISCUSSION

A remarkable proportion of patients included in the intermediate-risk cytogenetics group show diverse outcome. In the last decades, an effort has been made to identify novel molecular markers that could improve patient stratification in order to adapt their therapy to their prognosis. Up to now, only the analyses of NPM1, CEBPA and FLT3/ITD mutations have effectively been incorporated into routine prognostic stratification [6]. More recently, the mutations of RUNX1, TP53 and ASXL1 have been also associated with unfavorable outcome [4].

In the present study, we have identified for the first time, in two independent cohorts, that *NEDD9* overexpression is a favorable prognostic factor in intermediate-risk AML patients. This was, however, an unexpected finding since NEDD9 confers aggressiveness in most cancer types studied so far [14, 15]. A review of the NEDD9 published literature provided a rationale for our findings by identifying a distinct function for NEDD9 in normal and neoplastic myeloid cells as compared to normal lymphocytes or epithelial cells and their derived malignancies.

NEDD9 is a member of the CAS family of adhesion docking proteins. In adherent cells, NEDD9 regulates several signaling cascades involved in multiple activities including migration, adhesion, cell death or proliferation [18–20]. Because of the involvement of NEDD9 in key cellular functions, it is not surprising that a link has been established between its aberrant expression or activation and the aggressiveness or metastatic capacity of cancer cells [15]. Thus, overexpression of NEDD9 correlated with cell migration, invasion, metastasis development and drug resistance in several types of solid tumors such as glioblastoma, melanoma or breast cancer [19, 21].

However, NEDD9 shows opposite effects regarding migratory capacity on myeloid cells as compared to epithelial or lymphoid cells. Thus, NEDD9 blocks normal myeloid cell migration since NEDD9 deficient neutrophils [22], granulocytes or macrophages display increased migratory activity [23]. In sharp contrast, NEDD9 promotes migration in cultured normal human embryonic kidney cells [24] or fibroblasts [20], an effect occurring also in lymphocytes since NEDD9 deficient B cells show decreased migration as compared their wild type counterparts [22, 25]. The direction of the differences in normal cell migration between cells of the myeloid and lymphoid lineages observed *in vitro* is maintained in *in vivo* models. Thus, as compared to NEDD9+/+ mice, NEDD9 −/− mice show an increased number of macrophages, and a simultaneous reduction of B lymphocytes in peripheral blood [26] and secondary lymphoid organs, yielding an almost complete loss of Marginal Zone B (MZB) cells in the spleen [25].

The distinct NEDD9 functions observed in normal cells persist after neoplastic transformation, since NEDD9 overexpression blocks migration and dissemination of neoplastic cells of the myeloid lineage, while stimulating them in solid tumors or lymphoid neoplasias. Thus, NEDD9 inactivation associates with higher *in vivo* aggressiveness in chronic myeloid leukemia (CML), since NEDD9 deficient p210Bcr/Abl transgenic mice show an increased number of granulocytes in peripheral blood, a hyperplasia of myeloid and megakaryocytic cells in the bone marrow, and a diffuse myeloid infiltration in the spleen, lung and liver, leading to earlier progression and shorter mouse survival, which support NEDD9 capacity to block CML progression [23]. In contrast, NEDD9 mediates dissemination in BCR-ABL-dependent acute lymphoblastic leukemia (ALL) showing splenomegaly by accumulation of and pre-B cell lymphoblasts [21, 27]. Similarly, NEDD9 deficient p210Bcr/Abl mice show a significant decrease in lymphoid cell infiltration in bone marrow and spleen concomitantly with an increase in granulocytes [23]. In the same venue, in adult T-cell leukemia (ATL), the NEDD9 overexpression is induced by the Tax oncoprotein of the HTLV1 virus and enhances lymphocyte motility [28]. Finally, NEDD9 overexpression increases motility and/or induces epithelial-to-mesenchymal transition (EMT) in melanoma, glioblastoma and breast or colorectal carcinoma cells *in vitro* [29–32]. In addition, NEDD9 overexpression increases metastases and aggressiveness in *in vivo* models of these solid tumors [15, 19, 21, 33].

Based on the reports described above, the favorable prognosis we have observed for *NEDD9* overexpression in IR-AML patients is consistent with the notion that overexpression of *NEDD9* in AML cells may block their migratory and dissemination capacities, in contrast to the stimulation of *in vitro* migration or *in vivo* dissemination reported for cells on lymphoid or solid malignancies.

There are some limitations in our study. One of them is the lack of the CEBPA mutation analysis in the clinical data that lead to their exclusion in the univariate and multivariate analyses. However, CEBPA mutations are rather infrequent and because of that, they are unlikely to have an impact on the observed associations, despite an unequal distribution among high and low *NEDD9* expression groups. Another limitation is that the prognostic value of *NEDD9* cannot be extrapolated to patients older than 65 because they have not been included in this study. In addition, since minimal residual disease (MRD) status has been pointed out in recent studies as a strong prognostic factor for relapse and DFS in AML, it would be interesting to analyze the correlation between *NEDD9* expression and MRD status in the future. Unfortunately, the lack of MRD data for most of the studied patient cohort precluded this analysis. Nevertheless, our study is robust since *NEDD9* has been validated as a prognostic factor in two independent cohorts and has also been confirmed in the multivariate analyses.

In summary, our study identifies *NEDD9* overexpression as a good prognostic factor in IR-AML patients, because of its association with lower CIR, as well as with improved OS and DFS. If *NEDD9* overexpression is validated in future studies as an independent marker for a favorable outcome, it could be used for the development of risk-adapted post-remission chemotherapy protocols, to treat candidates with high probability of response among patients, included within the highly heterogeneous IR-AML subgroup. Finally, and because of the specificity of NEDD9 regulation and function in myeloid cells, it would be interesting to determine if the prognostic capacity we observed in AML patients for *NEDD9* overexpression could be extended to other myeloid lineage malignancies.

## MATERIALS AND METHODS

### Patients

Patients included in this study were adults up to the age of 65 years with *de novo* IR-AML, according to the Medical Research Council (MRC) classification [34, 35]. All the samples were collected at diagnosis after obtaining written informed consent in accordance with the Declaration of Helsinki and the Ethics Committee approval of each participating institution. A cohort of 73 patients (Cohort 1) from Hospital de la Santa Creu i Sant Pau (Barcelona, Spain) was our initial group, whereas another cohort of 206 patients from two other hospitals (Cohort 2), Hospital La Fe (Valencia, Spain) and Hospital Universitario de Salamanca (Salamanca, Spain), was included as a validation group in this study. All the patients were selected based on availability of bone marrow specimens, between the start and the end periods for each hospital trial. Clinical outcomes between the selected and non-selected patients of each cohort were similar and did not present significant differences (Data not shown). Patients older than 65 years or with an acute promyelocytic leukemia at diagnosis (M3 in FAB classification) were excluded from the study. The main characteristics of the groups are shown in Table 1.

Cohort 1 and Cohort 2 patients were treated according the CETLAM-03 (Grupo Cooperativo para el Estudio y Tratamiento de las Leucemias agudas y Mielodisplasias) and the PETHEMA trials LMA99, LMA2007 and LMA2010 (Programa de Estudio y Tratamiento de las Hemopatías Malignas, http://www.fundacionpethema.es/) protocols, respectively.

Concerning the CETLAM-03 protocol, treatment was administered between 2003 and 2012 at 21 collaborating institutions. In brief, induction chemotherapy included one or two courses of idarubicin 12 mg/m^2^ intravenously (IV) days 1, 3, 5, cytarabine 500 mg/m^2^/12 h over 2 h IV infusion days 1, 3, 5, and etoposide 100 mg/m^2^ IV days 1, 2, 3. In addition, patients also received G-CSF priming 150 mg/m^2^ subcutaneously (SC) from day 0 to the last day of induction and consolidation chemotherapy. This was followed by one consolidation with mitoxantrone 12 mg/m^2^ IV from day 4 to 6, and cytarabine 500 mg/m^2^/12 h IV from day 1 to 6. Subsequently, the patients went on chemotherapy courses with high-dose cytarabine as in the Cancer and Leukemia Group B trial [36]. Particularly, patients in intermediate risk group, with normal karyotype, a single course of induction chemotherapy to achieve the complete remission (CR), the absence of adverse molecular features (FLT3-ITD or MLL-PTD) and low minimal residual disease (MRD) levels after consolidation (MRD < 0,1%) receive an autologous peripheral blood stem cells (PBSC) transplant, regardless of having an HLA-identical sibling. The PETHEMA protocols are detailed in http://www.fundacionpethema.es/. Eligible patients were treated with intensive chemotherapy in which induction consisted of a combination of anthracycline plus cytarabine with or without etoposide. On achievement of complete remission (CR) patients proceeded to consolidation therapy and eligible cases were selected for autologous or allogeneic stem cell transplantation.

### Time-dependent clinical outcome endpoints

Overall survival (OS), disease-free survival (DFS) and cumulative incidence of relapse (CIR) were assessed in the statistical analysis. OS was calculated from the time of patient diagnosis to death or last date of follow-up (dead *vs* alive patients) and DFS from the complete remission date to relapse/death or last date of follow-up (dead or relapsed *vs* alive patients). Finally, CIR was calculated from the complete remission date to relapse or last date of follow-up or death (competing risk analysis: relapsed *vs* alive non-relapsed *vs* dead non-relapsed patients).

### RNA extraction and gene expression analyses

Samples were obtained from bone marrow aspirates of IR-AML patients at diagnosis. Mononuclear cells were isolated by Ficoll-Hypaque gradient and total RNA was extracted using TRIzol reagent (Thermo Fisher Scientific, Waltham, MA, USA) following the manufacturer's protocol. cDNA was generated after reverse transcription of 1.5 μg total RNA using the High Capacity cDNA Reverse Transcription Kit (Applied Biosystems (AB), Foster City, CA, USA). *NEDD9* and *BCAR1* expression (Hs00610590_m1 and Hs00183953_m1, resp. AB) was determined by real-time PCR using the platform ABI 7900HT Fast Real-Time PCR System (AB). Each sample was assessed by triplicate using the TaqMan Gene Expression Assay (AB). The comparative cycle threshold (DCt) method was used to determine relative expression levels [37], expressed as a ratio between target gene and control gene. *ABL* was used as the endogenous control gene (probe: ENP1043, primers: P3035: TGGAGATAACACTCTAAGCATAACTAAAGGT, P3036: GATGTAGTTGCTTGGGACCCA; AB) using VIC fluorophore. Quantitative methods analyses were detailed in previous reports also using *ABL* as control gene [38].

### Statistical analysis

Statistical analyses were performed in the IBM SPSS Statistics (Release 22.0.0.0, New York, NY, USA). The differences between clinical data of the two cohorts and the association between the *NEDD9* expression and clinical data were analyzed using the Fischer exact test or χ^2^ test. Differences between continuous variables were analyzed with the Student's *t*-test or the Mann-Whitney U test in those showing normal or abnormal distribution, respectively. Exploratory univariate analyses were performed to choose the threshold value to dichotomize the *NEDD9* and *BCAR1* mRNA levels in over and underexpression. After analyzing the mean, the median and different percentiles the best results were obtained with the third quartile (75th percentile) that is the cutoff selected to perform this study. Independent variables analyzed included age, gender, white blood cell count (WBC) at diagnosis, FAB category, and molecular alterations (FLT3/ITD, NPM1, and CEBPA). Time-dependent endpoints such as OS, DFS and CIR were assessed, and the endpoint for the threshold was death and relapse. Time-dependent outcomes were calculated using Kaplan–Meier curves (OS or DFS), and the log-rank test was used for comparisons [39]. The regression model used in univariate and multivariate analyses was COX test (OS and DFS). The free statistical package R Studio (Version 0.98-1102, 2009–2014, RStudio Inc., Boston, MA, USA) through the R version 3.1.2 (2014-10-31, The R Foundation for Statistical Computing) [40] was used for the competing risk analysis. CIR was analyzed by Gray [41] and Fine and Gray's tests [42], using R Studio and the *cuminc* and *crr* functions through the *cmprsk* package. After exploratory univariate comparisons, multivariate analyses were performed including variables with a *p-*value below 0.25 and the INTRO method was used in SPSS to analyze Cox regression. Hazard ratios (HR) with relative 95% confidence interval (CI) are shown in both univariate and multivariate analyses. Any differences were considered to be statistically significant when the *p-*value was < 0.05.

## SUPPLEMENTARY MATERIALS FIGURES AND TABLES



## Abbreviations

ABL: Abelson murine leukemia viral oncogene homolog 1
ALL: Acute Lymphoblastic Leukemia
AML: Acute Myeloid Leukemia
ATL: Adult T-cell Leukemia
BCAR1: Breast Cancer Anti-Estrogen Resistance 1
BCR: Breakpoint Cluster Region
CAS: Crk-Associated Substrate
CEBPA: CCAAT/enhancer-binding protein alpha
CETLAM: Grupo Cooperativo para el Estudio y Tratamiento de las Leucemias agudas y Mielodisplasias
CI: Confidence Interval
CIR: Cumulative Incidence of Relapse
CML: Chronic Myeloid Leukemia
CR: Complete Remission
CRK: Adaptor molecule Crk or p38
DFS: Disease-free survival
EMT: Epithelial-to-Mesenchymal Transition
FAB: French-American-British
FAK: Focal Adhesion Kinase
FLT3: Fms-related tyrosine kinase 3
FLT3/ITD: Fms-related tyrosine kinase 3/ Internal Tandem Duplication
HR: Hazard Ratio
HTLV1: Human T-cell Lymphotropic Virus type 1
IR-AML: Intermediate risk - Acute Myeloid Leukemia
IV: Intravenously
MLL: Mixed Lineage Leukemia
MRC: Medical Research Council
MRD: Minimal Residual Disease
MZB: Marginal Zone B
NEDD9: Neural precursor cell Expressed Developmentally Down-regulated protein 9
NPM1: Nucleophosmin 1
OS: Overall Survival
PBSC: Peripheral Blood Stem Cells
PETHEMA: Programa de Estudio y Tratamiento de las Hemopatías Malignas
Resp: Respectively
ROC: Receiver Operating Characteristic
RTK: Receptor Tyrosine Kinases
SC: Subcutaneously
SRC: Proto-oncogene tyrosine-protein kinase Src
WBC: White Blood Cells

## END NOTES

This article was written following the REMARK guidelines [43, 44].
