# Supplementary material for: NEDD9, an independent good prognostic factor in intermediate-risk acute myeloid leukemia patients
Source: Oncotarget. 2017 Jun 16;8(44):76003–14. doi: 10.18632/oncotarget.18537 (PMC5652681; doi:10.18632/oncotarget.18537)
Supplement: Supplementary file 1 [file oncotarget-08-76003-s001.pdf]

NEDD9, an independent good prognostic factor in intermediate-risk acute myeloid leukemia patients

SUPPLEMENTARY MATERIALS

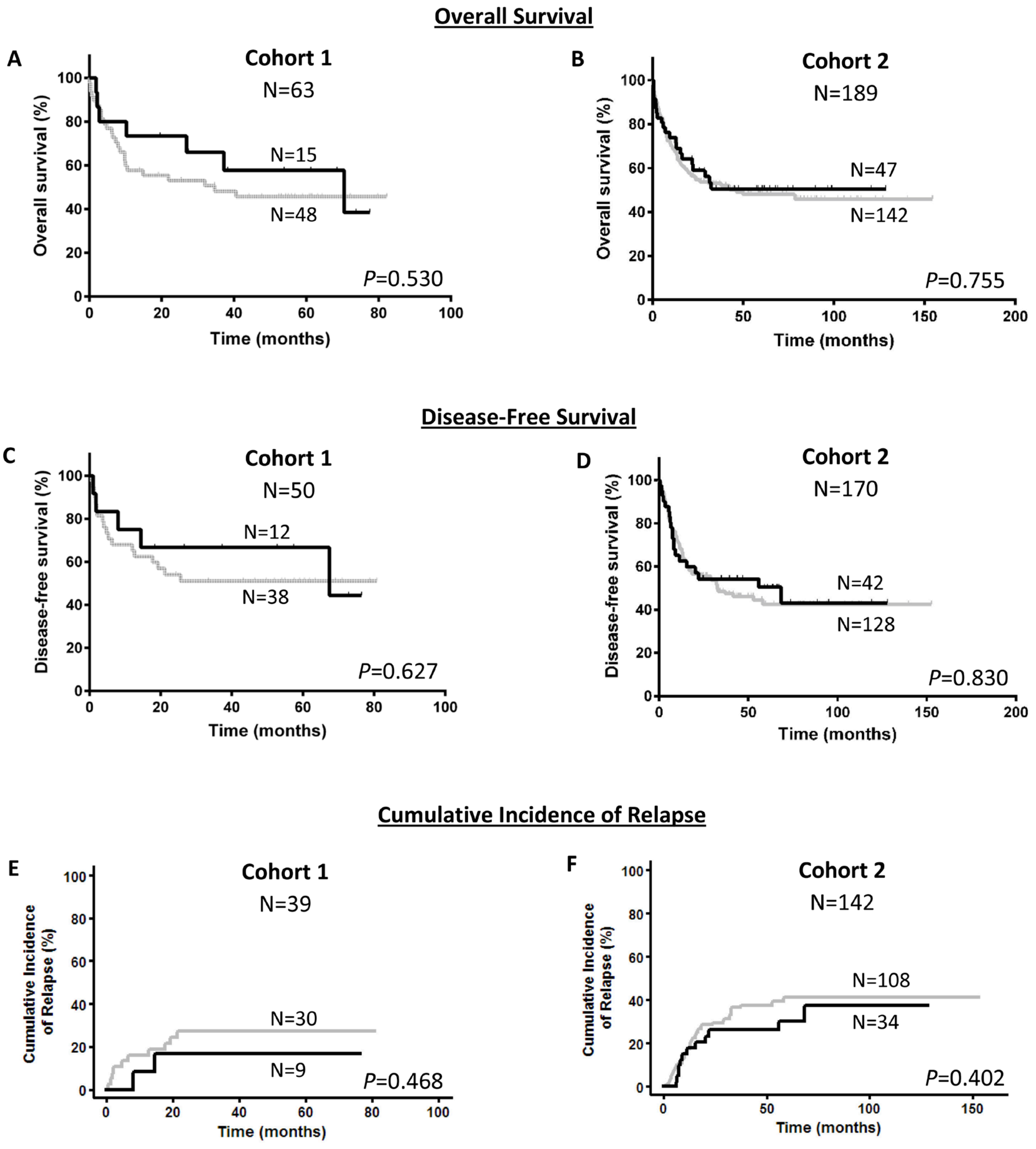

Supplementary Figure 1: OS, DFS and CIR according to *BCAR1* expression in both cohorts. Survival and cumulative incidence of relapse according to *BCAR1* expression in cohorts. Kaplan–Meier curves represent OS of cohort 1 (**A**) and cohort 2 (**B**), DFS of cohort 1 (**C**) and cohort 2 (**D**), CIR of cohort 1 (**E**) and cohort 2 (**F**), depending on the *BCAR1* expression. Black line and gray line indicate *BCAR1* overexpressed and underexpressed, respectively. Log-rank test for OS and DFS and Gray test for CIR were used to analyze the statistical significance. $P < 0.05$ was considered statistically significant (Bold values). OS; Overall survival. DFS; Disease-free survival. CIR; Cumulative incidence of relapse.

Supplementary Table 1: Time-dependent endpoints analyses regarding clinical characteristics of the cohorts of our study

	OS		DFS		CIR	
	**Cohort 1 (*n* = 73)**	**Cohort 2 (*n* = 206)**	**Cohort 1 (*n* = 59)**	**Cohort 2 (*n* = 185)**	**Cohort 1 (*n* = 59)**	**Cohort 2 (*n* = 185)**
Median of follow–up in months						
Alive (Range)	56 (3–82)	58 (1.7–154)				
Alive and CR patients (Range)			54 (2–81)	46 (0.1–153)	54 (2–81)	46 (0.1–153)
Survival, RR or CIR at 5 years						
(%)	47.0 ± 6.1	48.8 ± 3.7	51.4 ± 6.6	44.8 ± 4.1	29.6 ± 6.1	37.8 ± 3.9
Patient status						
Alive (%)	35 (48)	105 (51)	32 (54)	105 (57)	32 (54)	105 (57)
Death (%)	38 (52)	101 (49)	27 (46)	80 (43)	27 (46)	80 (43)
Relapsed (%)	17 (23)	64 (31)	17 (29)	64 (35)	17 (29)	64 (35)
Age, median (%)	***P* = 0.028**	***P* = 0.007**		***P* = 0.037**		
< 50 years	57.3 ± 8.7	58.7 ± 5.6	NS	52.6 ± 6.0	NS	NS
> 50 years	25.6 ± 11.8	38.1 ± 5.5	NS	32.1 ± 6.1	NS	NS
FLT3 duplication (%)				*P* = 0.024		
FLT3/ITD$^-$	NS	NS	NS	45.2 ± 5.3	NS	NS
FLT3/ITD$^+$	NS	NS	NS	32.9 ± 7.2	NS	NS
FLT3/NPM1 Combination (%)		***P* = 0.002**		***P* = 0.001**		***P* = 0.018**
FLT3$^-$ + NPM1$^+$ (Favourable)	NS	64.1 ± 8.2	NS	54.0 ± 9.0	NS	43.1 ± 4.9
Others (Unfavourable)	NS	40.3 ± 4.3	NS	36.6 ± 4.7	NS	35.1 ± 9.1

The results for the median follow-up are presented as the number of patients (range) for each condition; for the patient status as the number of patients (%); and for all the others as the estimates in percentage ± the standard error. Log-rank test was used to analyze the statistical significance for each variable. $P < 0.05$ was considered statistically significant (Bold values). NS represents not significant differences, and the estimates were not included in the table. CR; Complete remission. OS; Overall survival. DFS; Disease-free survival. CIR; Cumulative incidence of relapse.
